# The value of cardiac magnetic resonance imaging in endocardial fibroelastosis

**DOI:** 10.3389/fped.2022.874597

**Published:** 2022-11-01

**Authors:** Wenjiao Xiao, Yuanlin Wang, Weiqin Cheng, Yuting Zhang

**Affiliations:** Department of Radiology, Ministry of Education Key Laboratory of Child Development and Disorders, National Clinical Research Center for Child Health and Disorders, China International Science and Technology Cooperation Base of Child Development and Critical Disorders, Chongqing Key Laboratory of Pediatrics, Children’s Hospital of Chongqing Medical University, Chongqing, China

**Keywords:** pediatrics, endocardial fibroelastosis, cardiac function, cardiac magnetic resonance imaging, echocardiography

## Abstract

**Introduction:**

Endocardial fibroelastosis (EFE), an uncommon congenital heart disorder often occurring in infants, has a poor prognosis. It is of great significance to perform early diagnosis and accurately analyze cardiac function to enable further clinical treatment and prognosis decisions. This study aimed to explore the findings of cardiac magnetic resonance (CMR) in patients with EFE, including morphological changes and cardiac function analyses. Additionally, we compared the difference in the evaluation of the cardiac function between CMR and echocardiography (Echo).

**Methods:**

Eleven patients with EFE (nine females and two males, aged between 0.3 and 1.9 years), treated in our hospital, were analyzed retrospectively. Left ventricular posterior wall thickness (LVPW), anterior wall thickness (LVAW), fractional shortening (FS), ejection fraction (EF), end-systolic diameter (ESD), end-diastolic diameter (EDD), end-systolic volume (ESV), and end-diastolic volume (EDV) were assessed using both Echo and CMR. The Original Ross classification and the New York Heart Association functional classification were used to grade the patients**’** cardiac function. The correlations between clinical cardiac function classification and MRI- and Echo-derived imaging data were determined.

**Results:**

All patients showed a thickened endocardium and left ventricle globular dilatation on CMR. We observed significant systolic dysfunction and whole or segmental abnormal ventricular movement. Compared with those measured by Echo, the EF, FS, and EDV values were significantly lower when measured using CMR. Compared with Echo measurements, the ESV, ESD, LVAW, and LVPW values were significantly higher when measured using CMR. CMR-measured EF and FS correlated better with the clinical cardiac functional score than those derived from Echo (EF, *r* = 0.646 > 0.224; FS, *r* = 0.627 > 0.245, respectively).

**Conclusion:**

In patients with EFE, the characteristic morphological changes of the heart could be displayed accurately using CMR. The parameters measured by CMR were more accurate than those of Echo and correlated well with clinical cardiac function scores, mainly because it does not make invalid geometrical assumptions.

## Introduction

Collagen and elastic fiber hyperplasia in the endocardium results in endocardial thickening, a characteristic of endocardial fibroelastosis (EFE). Infants and young children more frequently develop EFE, but it can also present rarely in adulthood (1). Research showed that EFE might be related to viral infections, intrauterine hypoxia, immune factors, and genetic factors (2, 3). Clinical experience suggests that EFE mainly affects the systolic function of the left ventricle (LV) and manifests as severe congestive heart failure. Clinical findings mainly include heart failure, pulmonary infection, exercise intolerance, shortness of breath, and prolonged breastfeeding time (4). Importantly, the onset of EFE is rapid and patients might have a poor prognosis or even die without timely treatment (5, 6). Therefore, the diagnosis of EFE should be established early and accurately. The gold standard method to diagnose EFE is an endocardial biopsy. However, few people choose it because it is an invasive procedure (7). To date, EFE is usually diagnosed based on clinical features and echocardiography (Echo).

Although Echo is commonly performed for patients with EFE, research has shown that cardiac magnetic resonance (CMR) might be more visual and accurate in evaluating morphological and functional changes in patients’ hearts (8, 9). The higher contrast and resolution of CMR mean that it also provides excellent delineation of abnormal trabeculations. This permits the quantification and identification of the degree of endocardial thickening. This study aimed to explore the findings of CMR in patients with EFE and to examine its role in EFE diagnosis, including morphological changes and cardiac function analysis. Additionally, we determined the difference in the evaluation of cardiac function between CMR and Echo.

## Patients and methods

### Selection of patients

This was a retrospective study conducted on 11 patients with EFE. The patients received treatment at our hospital between November 2010 and July 2019. There were nine female and two male patients, aged between 0.3 and 1.9 years (average age = 0.9 ± 0.6). All patients underwent CMR and Echo within 2 weeks after being admitted to the hospital. The diagnosis of EFE was confirmed using clinical signs, Echo, and CMR using the diagnostic criteria of the World Health Organization/International Society and Federation of Cardiology Task Force (10). Two experienced pediatric radiologists with 10 years of expertise in cardiac imaging analyzed all images. The institutional review committee of our hospital approved the study, and informed consent was obtained from the patients’ parents or guardians.

Of the 11 patients, 8 presented with pneumonia, 4 presented with heart failure, and 2 presented with myocarditis. The Original Ross classification and the New York Heart Association functional classification were used to grade the patients’ cardiac function (Table 1) (11, 12). The classifications are as follows: one case of class I, two cases of class II, seven cases of class III, and one case of class IV.

**Table 1:** Clinical cardiac function classification of heart failure.

Grade	NYHA classification	Original Ross classification
I	Patients with cardiac disease but without resulting limitation of physical activity. Ordinary physical activity does not cause undue fatigue, palpitation, dyspnea, or anginal pain	No limitations or symptoms
II	Patients with cardiac disease resulting in slight limitation of physical activity. They are comfortable at rest. Ordinary physical activity results in fatigue, palpitation, dyspnea, or anginal pain	Mild tachypnea or diaphoresis with feedings in infants, dyspnea at exertion in older children; no growth failure
III	Patients with cardiac disease resulting in marked limitation of physical activity. They are comfortable at rest. Less than ordinary physical activity causes fatigue, palpitation, dyspnea, or anginal pain	Marked tachypnea or diaphoresis with feedings or exertion and prolonged feeding times with growth failure from congestive heart failure
IV	Patients with cardiac disease resulting in an inability to carry on any physical activity without discomfort. Symptoms of heart failure or anginal syndrome may be present even at rest. If any physical activity is undertaken, discomfort is increased	Symptomatic at rest with tachypnea, retractions, grunting, or diaphoresis

CMR, cardiac magnetic resonance; EFE, endocardial fibroelastosis.

### Protocols for CMR and the analysis of images

In this study, a 1.5 T MR scanner (Signa EXCITE HD; GE Healthcare, Chicago, IL, United States) incorporating a special eight-channel cardiac phased-array coil was used to carry out CMR measurements. An electrocardiography-gated technique and a respiratory-gated technique in real-time instances were used to avoid the influence of rhythm and breath. We obtained fast spin Echo with a double inversion recovery preparatory pulse and fast-imaging employing steady-state acquisition (FIESTA) cine images in one short-axis view and three long-axis views (two-, three-, and four-chamber) covering the LV from the base to the apex. Subsequently, 0.2 mmol/kg gadolinium-based contrast agent was injected intravenously. Ten minutes later, we performed myocardial delayed enhancement (MDE) using a T1-weighted inversion recovery gradient Echo sequence. Dedicated software (ReportCARD 3.0; GE Healthcare) was used by experienced radiology technicians to carry out the cardiac function analysis. In all scan slices, the endocardial and epicardial borders of the LV myocardium were traced manually at the end of diastole and systole on each cine short-axis image to determine LV fractional shortening (FS), LV ejection fraction (EF), LV end-systolic volume (ESV), and LV end-diastolic volume (EDV). At the end of the diastolic phase, we measured the left ventricular posterior wall thickness (LVPW) and left ventricular anterior wall thickness (LVAW). An ultrasound instrument (Vivid 7; GE Healthcare) using a probe frequency of 2.5 MHz was used to carry out Echo measurements. An EchoPAC workstation (GE Vingmed Ultrasound AS, Horten, Norway) was used for data analysis.

### Statistical analyses

The data were consistent with the skewness distribution and were described by the median and interquartile range (IQR). Statistical differences were tested using the Wilcoxon signed-rank test. Correlations between clinical cardiac functions and CMR/Echo characteristics were calculated using Spearman’s rank correlation analysis. Statistically significant differences were identified by a *P* value < 0.05. SPSS version 25.0 (IBM Corp., Armonk, NY, United States) was used to conduct statistical analyses.

## Results

**Figure 1:**
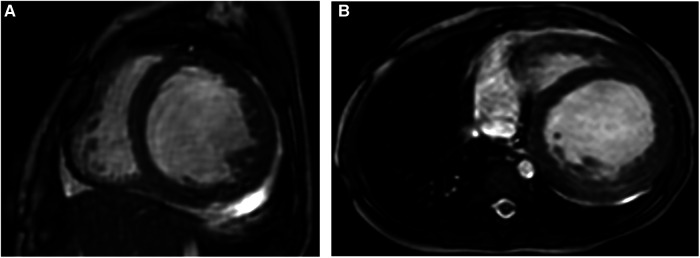
Representative MRI scans of a 1-year-old female patient with EFE who presented with severe heart failure and had PCR once due to the heart event. (**A,B**) Short-axis and axial FIESTA cine showing thickened endocardium with obviously increased and hypertrophied myocardial trabeculae. EFE, endocardial fibroelastosis.

**Figure 2:**
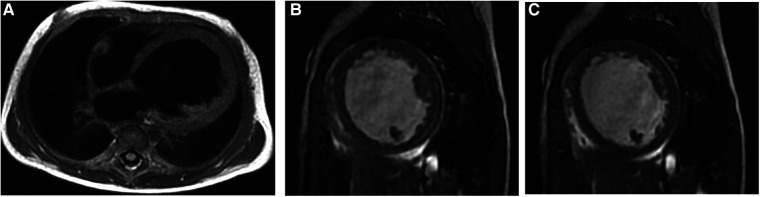
Representative MRI scans of a 5-month female EFE patient who presented with cyanosis and shortness of breath. (**A**) Four-chamber D-IR FSE showing LV globular enlargement; the ventricular septum is obviously protruding to the right ventricular side. (**B,C**) Short-axis FIESTA cine at the end of systole and diastole, respectively, showing thickened endocardium and reduced movement of the LV. EFE, endocardial fibroelastosis; LV, left ventricle.

**Figure 3:**
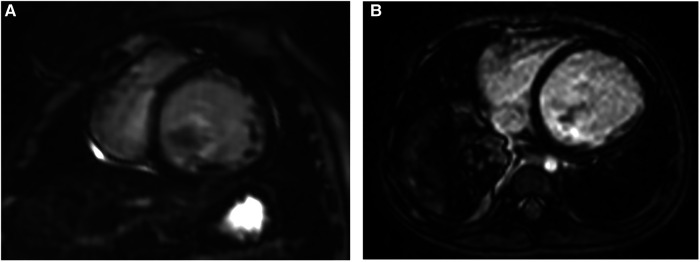
Representative images of MDE in two different EFE patients. (**A,B**) Short-axis and axial views showing no obvious delayed enhancement in both. EFE, endocardial fibroelastosis.

In all cases, CMR showed a thickened endocardium (mostly larger than 2–3 mm) with increased and hypertrophied myocardial trabeculae. The boundary between the thickened myocardial trabeculae and the compact myocardium was clear, which was located in the inferior wall, posterior wall, and posterior ventricular septum of the left ventricle. In the short-axis position, the lesion range was more than one-third or one-half of the circumferential diameter (Figure 1). The left ventricle was globularly dilatated, and the ventricular septum was obviously protruding to the right ventricular side, with reduced EF and FS. Moreover, we observed whole or segmental abnormal ventricular movement (Table 2, Figure 2). Among our patients, four received MDE, which revealed no obvious endocardial enhancement in any of them (Figure 3).

**Table 2:** Morphological findings of CMR in 11 patients with EFE.

Patient	Age (year)	Globularly dilatated left ventricle^a^ (mm)	Whole or segmental decreased ventricular wall movement^b^ (%)	Location of myocardial involvement^c^	Lesion range of myocardial involvement^d^
1	0.5	40	11	Anterior, lateral, and posterior walls of the left ventricle	1/2
2	0.3	32	23.9	Posterior ventricular septum, anterior, lateral, posterior, and inferior walls of the left ventricle	2/3
3	1.8	36	14.3	Posterior and inferior wall of the left ventricle	1/3
4	0.4	51	13.8	Anterior ventricular septum, anterior, lateral, and posterior walls of the left ventricle,	1/2–2/3
5	1.9	53	14.8	Lateral, and posterior walls of the left ventricle	1/3
6	0.3	46	15.8	Lateral, posterior, and inferior walls of the left ventricle	1/2
7	1	46	14.8	Posterior ventricular septum, anterior, lateral, posterior, and inferior walls of the left ventricle	2/3
8	1.4	48	15.7	Anterior, lateral, posterior, and inferior walls of the left ventricle	2/3
9	1.2	44	11.1	Lateral and posterior walls of the left ventricle	1/3
10	0.9	40	12.3	Anterior, lateral, and posterior walls of the left ventricle	1/2
11	0.4	30	29.6	Lateral, posterior, and inferior walls of the left ventricle	1/2

CMR, cardiac magnetic resonance; EFE, endocardial fibroelastosis.

^a^ Described by the left ventricular end-diastolic diameter.

^b^ Described by ejection fraction.

^c^ CMR showed thickened endocardium and obviously increased and hypertrophied myocardial trabeculae.

^d^ The lesion range of the circumferential diameter in the short-axis section of the papillary muscle.

**Figure 4:**
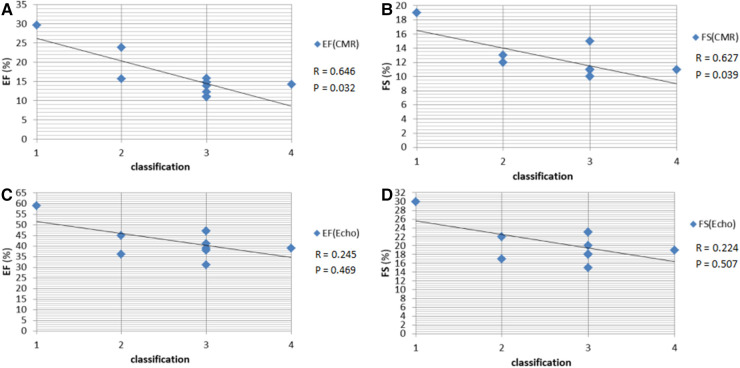
(**A,B**) Scatterplot of clinical cardiac function classification and EF and FS measured by CMR in patients with EFE. (**C,D**) Scatterplot of clinical cardiac function classification and EF and FS measured by Echo in patients with EFE. EF, ejection fraction; FS, fractional shortening; CMR, cardiac magnetic resonance; EFE, endocardial fibroelastosis.

The data for all 11 patients, including their clinical cardiac functional score and their measured EF and FS (based on CMR vs. Echo), are shown in Table 3. The LV function indexes measured by CMR and Echo in patients with EFE were analyzed. The values of EF, FS, and EDV were lower when measured using CMR than when measured using Echo. Compared with those in the Echo group, the values of ESV, ESD, LVAW, and LVPW were significantly higher in the CMR group (*P* < 0.05; Table 4). There was no significant difference for end-diastolic diameter (EDD). CMR-determined EF and FS correlated well with the classification of clinical cardiac function (*r* = 0.646 and *r* = 0.627, respectively). Meanwhile, Echo-classified EF and FS also correlated with clinical cardiac function classes (*r* = 0.224 and *r* = 0.245, respectively) (Figure 4).

**Table 3:** Clinical cardiac function classification and EF and FS measured by CMR or Echo.

Patient	Age (year)	CMR		Echo		Classification
		EF (%)	FS (%)	EF (%)	FS (%)	
1	0.5	11	10	39	18	III
2	0.3	23.9	13	45	22	II
3	1.8	14.3	11	39	19	IV
4	0.4	13.8	11	31	15	III
5	1.9	14.8	15	41	20	III
6	0.3	15.8	11	38	18	III
7	1	14.8	11	47	23	III
8	1.4	15.7	12	36	17	II
9	1.2	11.1	11	47	23	III
10	0.9	12.3	10	38	18	III
11	0.4	29.6	19	59	30	I

EF, ejection fraction; FS, fractional shortening; CMR, cardiac magnetic resonance.

**Table 4:** LV parameters measured by CMR and Echo in patients with EFE.

Technique	EF (%)	FS (%)	EDV (ml)	ESV (ml)	EDD (mm)	ESD (mm)	LVAW (mm)	LVPW (mm)
CMR	14.8 (15.8–12.3)	11.0 (13.0–11.0)	59.6 (75.1–51.1)	50.8 (67.3–45.4)	44.0 (48.0–36.0)	40.0 (44.0–32.0)	5.0 (6.0–5.0)	5.0 (6.0–4.0)
Echo	39.0 (47.0–38.0)	19.0 (23.0–18.0)	78.5 (107.5–47.4)	44.1 (65.9–27.0)	42.0 (48.0–34.0)	33.0 (39.0–27.0)	4.0 (5.0–3.0)	5.0 (5.0–3.0)
*P*-value	0.003	0.003	0.008	0.041	0.240	0.029	0.031	0.034

CMR, cardiac magnetic resonance; EFE, endocardial fibroelastosis; EF, ejection fraction; FS, fractional shortening; EDV, end-diastolic volume; ESD, end-systolic diameter; EDD, end-diastolic diameter; ESD, end-systolic diameter; LVAW, left ventricular anterior wall thickness; LVPW, left ventricular posterior wall thickness.

Note: Data were described by the median and interquartile range.

## Discussion

Left ventricular dysfunction caused by EFE sometimes causes fulminant progression to heart failure. Early diagnosis, early treatment, and long-term adherence are the key to a good prognosis (13). Histological examination remains the “gold standard” for EFE diagnosis. However, because of its noninvasive nature, lack of radiation, and economy, currently, Echo is the most frequently used technique to diagnose EFE clinically in our country. However, CMR has high spatial and tissue resolution that can accurately observe the morphological changes of the heart and analyze cardiac function (8, 9). The 11 cases in our study were assessed using both CMR and Echo within 2 weeks. Our findings showed that in pediatric patients with EFE, CMR could visualize morphological changes accurately and detect functional alterations. By contrast, Echo-based diagnosis of EFE has limited sensitivity and accuracy because of its small field of vision, low spatial resolution, and strong dependence on the experience of the person carrying out the Echo. Additionally, MDE has been reported as a method to represent fibrosis of the myocardium (14). In typical cases, a rim of hyperintense signal in the myocardium will be found in MDE images (15). However, in our study, only four patients received MDE and none of them had obvious delayed enhancement. This might be related to the insufficient delayed time from contrast agent injection to scanning. Research showed that the best discrimination of the enhancement of EFE was noted 15–20 min after contrast agent injection (15). Further research is needed to improve this area.

Additionally, for patients who are suspected of having EFE, it is sometimes difficult to distinguish EFE from left ventricular noncompaction (LVNC). The best method of diagnosis is endocardial biopsy, which is an invasive method and is not recommended. CMR can clearly show the border of the endocardium and the compact myocardium, which can be used to distinguish these two diseases. Deep trabecular fossae communicating within the LV cavity and multiple prominent trabeculae in the subendocardial area characterize LVNC. LVNC can also be manifested as apparent thickening of the endocardium, similar to EFE. The difference is that the compacted myocardium is significantly thinned in LVNC, but not in EFE (16). The left ventricular wall thickness was normal in all our cases (average LVPW: 5.18 ± 1.25 mm). Additionally, dilated cardiomyopathy (DCM) and EFE both result in an enlarged heart and manifest as congestive heart failure. DCM is mainly characterized by the enlargement of bilateral cardiac chambers, along with normal or thin myocardial wall thickness, without endocardial thickening (17). Mural thrombus and regional wall motion abnormalities can be found in some cases of DCM (18). Furthermore, patients with EFE are significantly younger than patients with DCM (19).

Compared with Echo, CMR can display the heart more comprehensively and evaluate cardiac function more accurately. CMR can scan the entire heart and calculate the ejection fraction by delineating the endocardium and epicardium layer by layer with good repeatability, while Echo mainly evaluates it using geometric formulas. A cine sequence on CMR can demonstrate well the reduced movement of the left ventricle wall. However, Echo might be affected by the proficiency of the operator and the acoustic window. In the analysis of cardiac function, the LV function measured by Echo is based on the LV geometry and the selection of the most appropriate formula to calculate the LV volume. Therefore, Echo might overestimate the cardiac function of patients with EFE and the severity of the disease could be missed. The results of this study demonstrated that the LV-associated function indexes (i.e., EDV, EF, and FS) were significantly lower in the CMR group than in the Echo group (*P* < 0.05). Compared with those of Echo, CMR-determined EF and FS correlated better with the clinical cardiac functional score (EF, *r* = 0.646 > 0.224; FS, *r* = 0.627 > 0.245, respectively). Echo does have some advantages. It is a portable technique and is rapid and cheap. Moreover, Echo is often the first choice for most patients. However, with the development of magnetic resonance imaging (MRI) and the use of electrocardiography and respiratory gating, cardiac MRI can play a more important role in diagnosing EFE. Patients suspected of having EFE should be recommended to receive a CMR for diagnosis and a comprehensive evaluation of their cardiac function (20, 21).

Additionally, we plan to include some progressing techniques, such as T1 mapping, to quantitatively analyze myocardial tissue in a future study. T1 mapping is a quantitative assessment of myocardial tissue characteristics involving measuring the myocardial T1 value and the extracellular volume fraction (22). It is a noninvasive technique that can be used to detect diffuse myocardial fibrosis of some myocardial diseases, such as DCM; however, to date, there has been no report on its application in endocardial fibroelastosis (23).

In our study, we found that the bright-blood cine sequence had the most diagnostic value. First, a globularly dilatated left ventricle and the whole or segmental decreased ventricular wall movement could be observed on it. The most helpful finding is that with dynamic observation on the bright-blood cine sequence, the thickened endocardium with obviously increased and hypertrophied myocardial trabeculae could be identified at the end of diastole and, from the aggregate myocardial trabeculae, which resembled a “thickened myocardium,” at the end of systole, which might be misdiagnosed as hypertrophic cardiomyopathy (HCM). Additionally, the boundary between the thickened myocardial trabeculae and the compact myocardium was clear, which can distinguish EFE from LVNC.

## Study limitations

This study had some limitations. First, this was a single-center retrospective study. Second, the number of cases was relatively low. Therefore, it is necessary to expand the number of samples for further research.

## Conclusions

CMR can accurately display the characteristic morphological changes of the heart in patients with EFE. In the analysis of cardiac function, the values measured by CMR were more accurate than those measured using Echo and had a good correlation with clinical cardiac function. Of course, CMR also has some limitations such as long examination times and higher costs. With the further development of medical technology, improvements in CMR can be expected in the future.

## Data Availability

The raw data supporting the conclusions of this article will be made available by the authors, without undue reservation.

## References

[1] Steger CM Antretter H Moser PL. Endocardial fibroelastosis of the heart. Lancet. (2012) 379(9819):932. 10.1016/S0140-6736(11)61418-9 22217671

[2] Sana MK, Mahajan K. Endocardial fibroelastosis. [Updated 2022 Jun 5]. In: *StatPearls [Internet]*. Treasure Island, FL: StatPearls Publishing (2022). Available from: https://www.ncbi.nlm.nih.gov/books/NBK559128/32644554

[3] Aoki H Inamura N Kawazu Y Nakayama M Kayatani F. Fetal echocardiographic assessment of endocardial fibroelastosis in maternal anti-SSA antibody-associated complete heart block. Circ J. (2011) 75(5):1215–21. 10.1253/circj.CJ-10-1032 21436591

[4] Ino T, Benson LN, Freedom RM, Rowe RD. Natural history and prognostic risk factors in endocardial fibroelastosis. *Am J Cardiol*. (1988) 62(7):431–4. 10.1016/0002-9149(88)90972-1 3414520

[5] De Letter EA Piette MH. Endocardial fibroelastosis as a cause of sudden unexpected death. Am J Forensic Med Pathol. (1999) 20(4):357–63. 10.1097/00000433-199912000-00009 10624930

[6] Takahashi S Kanetake J Moriya T Funayama M. Sudden infant death from dilated cardiomyopathy with endocardial fibroelastosis. Leg Med (Tokyo). (2008) 10(5):277–80. 10.1016/j.legalmed.2008.03.001 18442941

[7] Chan JL Rosing DR Klion AD Horvath KA. Surgical management of adult endocardial fibroelastosis. J Thorac Cardiovasc Surg. (2017) 154(5):e81–4. 10.1016/j.jtcvs.2017.05.050 28668457 PMC6341226

[8] Zhang Y He L Cai J Lv T Yi Q Xu Y Measurements in pediatric patients with cardiomyopathies: comparison of cardiac magnetic resonance imaging and echocardiography. Cardiology. (2015) 131(4):245–50. 10.1159/000381418 25969374

[9] Ziółkowska L Śpiewak M Małek Ł Boruc A Kawalec W. The usefulness of cardiovascular magnetic resonance imaging in children with myocardial diseases. Kardiol Pol. (2015) 73(6):419–28. 10.5603/KP.a2014.0239 25563465

[10] Richardson P Mekenna W Bristow M Maisch B Mautner B O'Connell J Report of the 1995 world health organization/international society and federation of cardiology task force on the definition and classification of cardiomyophathies. Circulation. (1996) 93(5):841–2. 10.1161/01.CIR.93.5.841 8598070

[11] The Criteria Committee of the New York Heart Association. Functional capacity and objective assessment. In: Dolgin M, editors. Nomenclature and criteria for diagnosis of diseases of the heart and great vessels. 9th ed. Boston, MA: Little, Brown and Company (1994). p. 253–5.

[12] Ross RD Bollinger RO Pinsky WW. Grading the severity of congestive heart failure in infants. Pediatr Cardiol. (1992) 13(2):72–5. 10.1007/BF00798207 1614922

[13] Oh NA Hong X Doulamis IP Meibalan E Peiseler T Melero-Martin J Abnormal flow conditions promote endocardial fibroelastosis via endothelial-to-mesenchymal transition, which is responsive to losartan treatment. JACC Basic Transl Sci. (2021) 6(12):984–99. 10.1016/j.jacbts.2021.10.002 35024504 PMC8733675

[14] Franco A Javidi S Ruehm SG. Delayed myocardial enhancement in cardiac magnetic resonance imaging. J Radiol Case Rep. (2015) 9(6):6–18. 10.3941/jrcr.v9i6.2328 26622933 PMC4638378

[15] Stranzinger E Ensing GJ Hernandez RJ. MR Findings of endocardial fibroelastosis in children. Pediatr Radiol. (2008) 38(3):292–6. 10.1007/s00247-007-0707-7 18172637

[16] Zuccarino F Vollmer I Sanchez G Navallas M Pugliese F Gayete A. Left ventricular noncompaction: imaging findings and diagnostic criteria. AJR Am J Roentgenol. (2015) 204(5):W519–30. 10.2214/AJR.13.12326 25905958

[17] Seki A Patel S Ashraf S Perens G Fishbein MC. Primary endocardial fibroelastosis: an underappreciated cause of cardiomyopathy in children. Cardiovasc Pathol. (2013) 22(5):345–50. 10.1016/j.carpath.2013.02.003 23518027

[18] D’Anastasi M Greif M Reiser MF Theisen D. MRT Bei dilatativen Kardiomyopathien [Magnetic resonance imaging of dilated cardiomyopathy]. Radiologe. (2013) 53(1):24–9 (in German). 10.1007/s00117-012-2382-4 23338246

[19] Luca AC Lozneanu L Miron IC Trandafir LM Cojocaru E Pădureţ IA Endocardial fibroelastosis and dilated cardiomyopathy—the past and future of the interface between histology and genetics. Rom J Morphol Embryol. (2020) 61(4):999–1005. 10.47162/RJME.61.4.02 34171049 PMC8343576

[20] Alperi A Martín M Vigil-Escalera M Silva I Cigarrán H Morís C. Young male with long-term impaired ejection fraction—endocardial fibroelastosis: the importance of cardiac magnetic resonance. Arch Cardiol Mex. (2019) 89(1):167–8 (in English). 10.24875/ACME.M19000040 31702740

[21] Raman SV Mehta R Walker J Pennell DJ. Cardiovascular magnetic resonance in endocardial fibroelastosis. J Cardiovasc Magn Reson. (2005) 7(2):391–3. 10.1081/JCMR-200053548 15881519

[22] Puntmann VO Peker E Chandrashekhar Y Nagel E. T1 mapping in characterizing myocardial disease: a comprehensive review. Circ Res. (2016) 119(2):277–99. 10.1161/CIRCRESAHA.116.307974 27390332

[23] Everett RJ Stirrat CG Semple SI, Newby DE, Dweck MR, Mirsadraee S. Assessment of myocardial fibrosis with T1 mapping MRI. Clin Radiol. (2016) 71(8):768–78. 10.1016/j.crad.2016.02.013 27005015

